# Dataset on the phytochemicals, antioxidants, and minerals contents of pecan nut cake extracts obtained by ultrasound-assisted extraction coupled to a simplex-centroid design

**DOI:** 10.1016/j.dib.2019.105095

**Published:** 2020-01-07

**Authors:** Laércio Galvão Maciel, Gerson Lopes Teixeira, Jane Mara Block

**Affiliations:** Department of Food Science and Technology, Federal University of Santa Catarina, 88034-001, Florianópolis, SC, Brazil

**Keywords:** *Carya illinoinensis*, Green extraction, Mixture design, Coefficients of regression, Optimization

## Abstract

This article contains a dataset related to the research published in “The potential of the pecan nut cake as an ingredient for the food industry” [1]. A three-component simplex-centroid mixture design coupled with response surface methodology (RSM) was applied to generate statistical models and to analyze the dataset. The method was also applied to evaluate the effect of different solvents (ethanol, water, and acetic acid) on the extraction of bioactive compounds of pecan nut cake (PNC) and its antioxidant activity. Furthermore, simultaneous optimization of the solvent mixture was carried out to predict the optimum point with the best combination of solvents to obtain an extract with enhanced phytochemical composition, as well as high *in vitro* antioxidant activity. The maximization of total phenolic compounds, condensed tannins, and antioxidant activity of the PNC was predicted by the desirability function. A total of 80 interactions were run to provide the best condition for optimization. The combined use of the different solvents enables a higher recovery of the compounds than their isolated use. This dataset may help other researchers on the application of a mixture design to recover phytochemicals from a broad range of co-products such as defatted meals and other nut cakes, which are sometimes discarded as waste by many industries.

**Table:** Specifications Table

Subject	Food Science
Specific subject area	Agricultural and Biological Sciences
Type of data	Tables, figures, text file
How data were acquired	Ultrasound-Assisted-Extractions (UAE) were performed on an EGS-5HD Ultrasonic system (Enge Solutions, São Paulo, Brazil) at 40 kHz and 300 W. The data related to the phytochemicals and antioxidant activity of UAE were acquired from different assays and calculated from the absorbances measured on a microplate reader (Spectramax Paradigm, Molecular Devices, San Jose-CA, USA), and analyzed using Statistica v. 10.0 (StatSoft Inc., USA), Microsoft Office Excel® v. 2016 (Microsoft Inc., USA) and Action v.2.9 (Statcamp, Brazil). An Analyst 200 equipment (PerkinElmer Inc., Waltham, EUA) and A 910 M flame photometer (Analyzer Comércio e Indústria Ltda., São Paulo, Brazil) were used to identify and quantify the minerals.
Data format	Raw, Analyzed
Parameters for data collection	The flame atomic absorption spectrometry (F-AAS) and Flame Atomic Emission Spectrometry (F-AES) were used to determine trace elements and minerals in the pecan nut cake. The simplex-centroid mixture design (cubic model), composed of 10 trials, was used to establish the experimental conditions for the *in vitro* analysis.
Description of data collection	For the quantification of minerals, the absorbances were recorded at different wavelengths as follows: λ = 422.67 (Ca), 324.80 (Cu), 240.73 (Co), 213.86 (Zn), 285.21 (Mg), 279.50 (Mn) Iron 248.30 (Fe), 589.00 (Na) and 710.00 (K). A Design of Experiments (DOE) was used in combination with the response surface methodology. Regression equations related to the independent variables (ethanol, water, and acetic acid) with the responses (phytochemicals and antioxidant activity) were obtained. These data were used to predict the quantitative value of each response, within the range of the tested values, to values (of independent factors) not tested in the experiment.
Data source location	Oils and Fats Laboratory, at the Department of Food Science and Technology, Federal University of Santa Catarina, Florianópolis, Santa Catarina, Brazil.
Data accessibility	The data are available in this article.
Related research article	Maciel, L.G., Ribeiro, F.L., Teixeira, G.L., Molognoni, L., dos Santos, J.N., Nunes, I.L., & Block, J.M. (2020). The potential of the pecan nut cake as an ingredient for the food industry. Food Research International, 127, 108718. DOI: https://doi.org/10.1016/j.foodres.2019.108718

**Table:** 

**Value of the Data** • Data show that non-toxic organic solvents as ethanol, water, and acetic acid may be used as an alternative to increase the yield of phenolic compounds and the antioxidant activity of extracts obtained from the pecan nut cake in an easy approach aided by ultrasound-assisted extraction and the simplex centroid design. Thus, the applied mathematical and statistical tools were able to provide an optimized extraction method and generate mathematical models with satisfactory prediction capability, which may be useful in the extraction of other raw materials. • Optimized values obtained from the desirability function of the extraction process offer support and may help other researchers on the recovery of bioactive compounds from different by-products. The dataset should also encourage the use of raw materials that are usually considered waste as an ingredient in the food, feed, pharmaceutical, and cosmetics industries adding value to them. • The multi-response analytical optimization was shown to be a feasible strategy to improve the process conditions and to obtain a product with unique characteristics. Thus, the presentation of the statistical tools used herein is intended to support not only pecan nut researchers, but also professionals of food science and technology, microbiology, food development, sensory evaluation, and nutrition, promoting a reduction in time, labor and operations costs. • The dataset presents information and tools that help the researchers to estimate the influence of variables on extraction processes by rejecting the variables that do not seem to contribute to the quality of the final product and to optimize the process conditions in order to obtain an improved extract.

## 1 Data

The pecan nut (*Carya illinoinensis* (Wang.) K. Koch) cake (PNC) is a by-product of the pecan nut oil extraction, which is rich in bioactive compounds. Therefore, this fraction has the potential to be used for the extraction of such substances [1]. The presented dataset shows a statistical approach on the extraction procedure employed for establishing the influence of different non-toxic solvents on the phytochemicals (total phenolic compounds - TPC, and condensed tannins - CT) and antioxidant activity (reducing potential of the hydrophilic compounds – RPHC, 2,2-diphenyl-1-picrylhydrazyl - DPPH, and total reducing capacity – TRC) of PNC, as well as for the quantification of minerals in PNC.

**Fig. 1:**
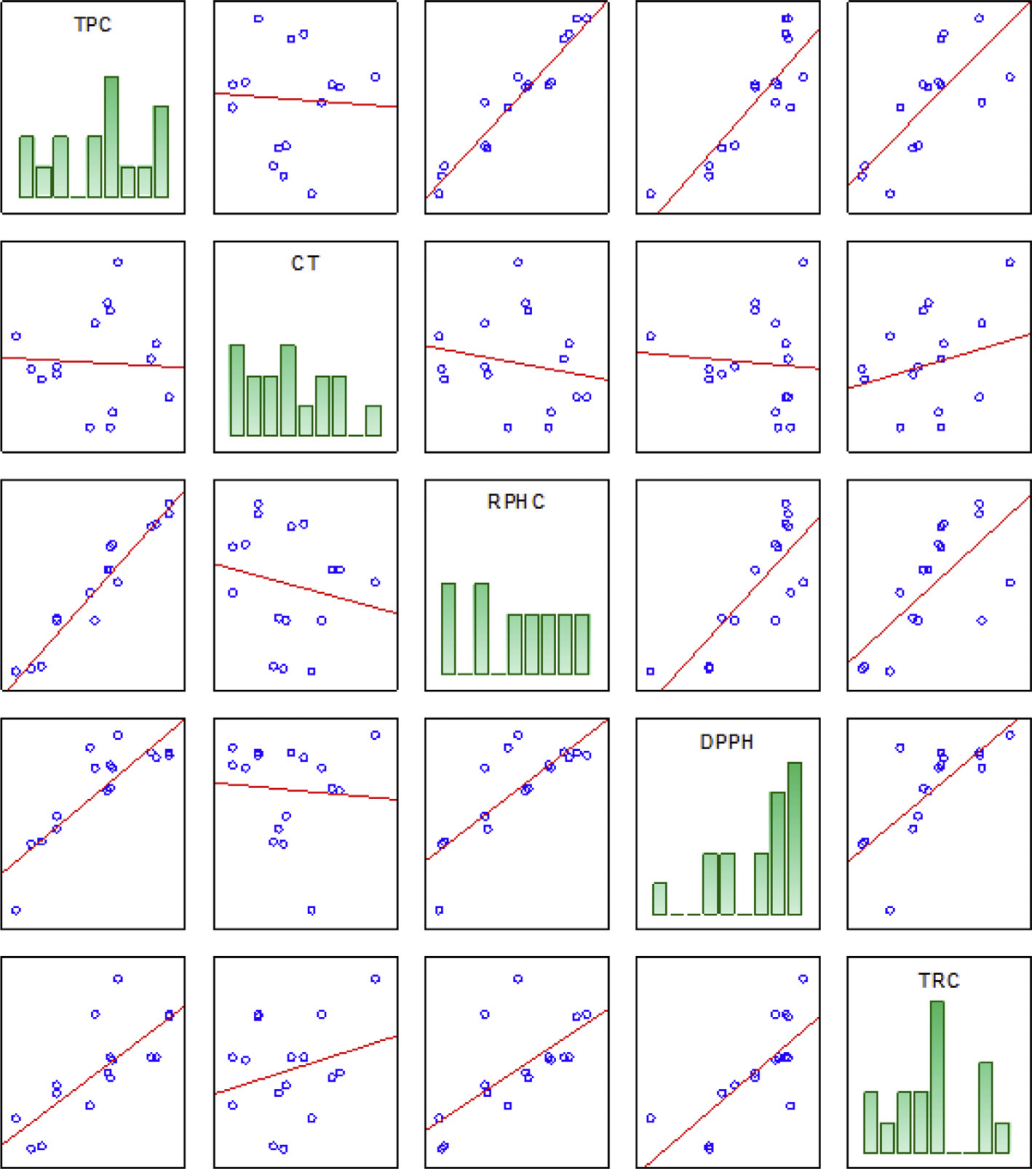
**Correlation analysis between the content of phenolics, condensed tannins, and antioxidant activity of the pecan nut cake extracts.** TPC: Total phenolic compounds; CT: Condensed tannins; RPHC: Reducing potential of the hydrophilic compounds; TRC: Total reducing capacity.

**Fig. 2:**
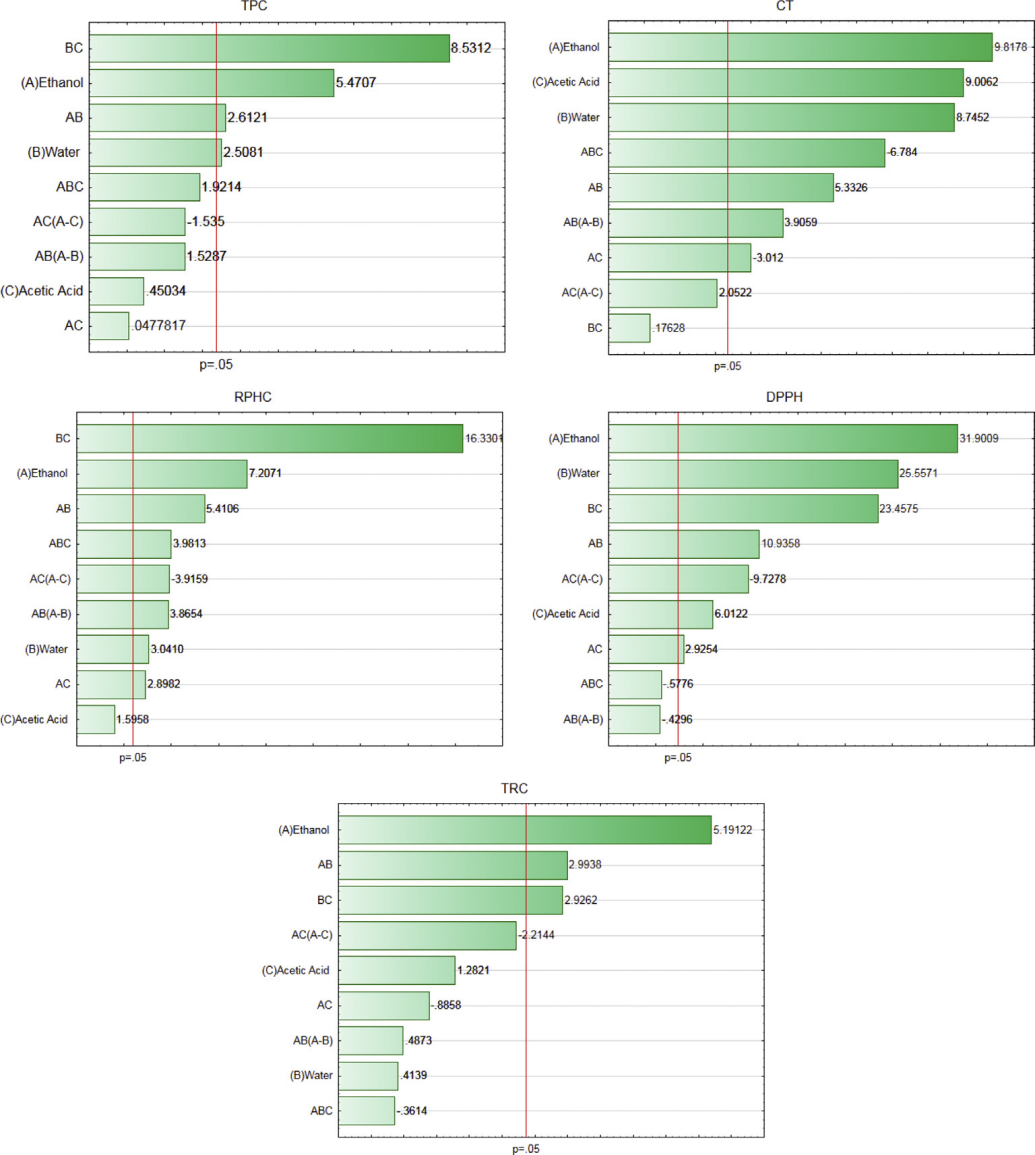
**Raw Pareto charts (not adjusted) showing the effects of the solvents (ethanol, water, and acetic acid) on the extraction of phytochemicals and the antioxidant activity of pecan nut cake.** TPC: Total phenolic compounds; CT: Condensed tannins; RPHC: Reducing potential of the hydrophilic compounds; TRC: Total reducing capacity; AB, AC, BC, and ABC are interaction effects.

**Fig. 3:**
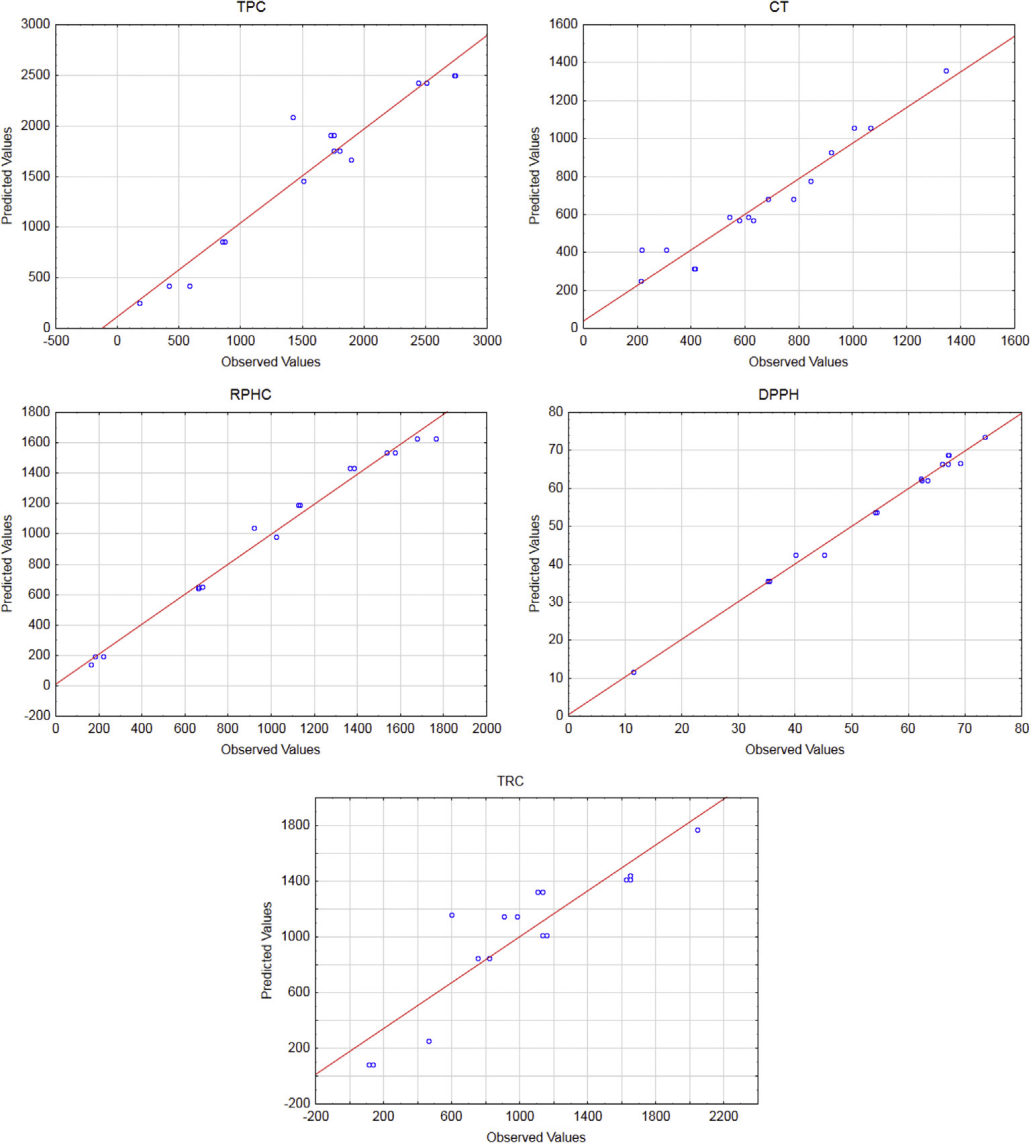
**Normal probability plot of residual values for the mixture of solvents (ethanol, water, and acetic acid) on the extraction of phytochemicals and the antioxidant activity of pecan nut cake.** TPC: Total phenolic compounds; CT: Condensed tannins; RPHC: Reducing potential of the hydrophilic compounds; TRC: Total reducing capacity; AB, AC, BC, and ABC are interaction effects.

**Fig. 4:**
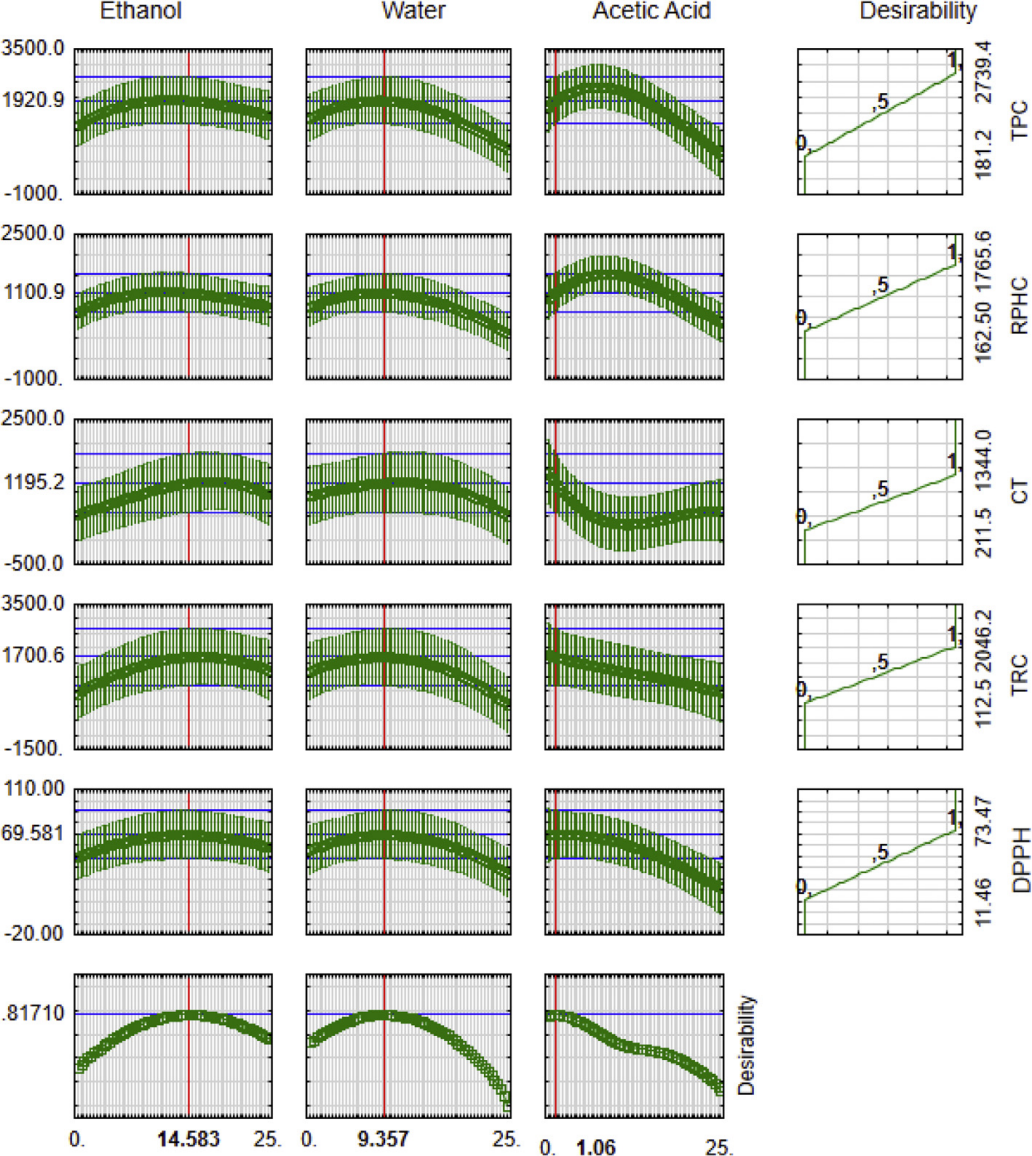
**Multi-response optimization of the mixture of solvents to maximize the total phenolic content and antioxidant activity of the pecan nut cake.** TPC: Total phenolic compounds; CT: Condensed tannins; RPHC: Reducing potential of the hydrophilic compounds; TRC: Total reducing capacity.

**Fig. 5:**
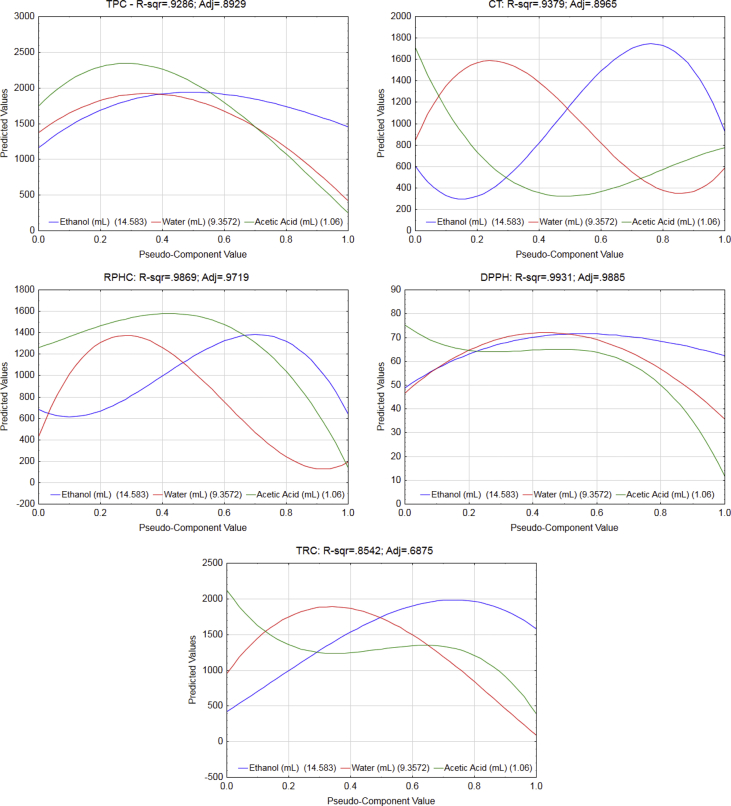
**Trace graph of the expected responses of solvents mixture.** TPC: Total phenolic compounds; CT: Condensed tannins; RPHC: Reducing potential of the hydrophilic compounds; TRC: Total reducing capacity; Coefficient of determination R-sqr: R-squared or R^2^ (regression coefficient) and R-adj: adjusted R^2^.

**Fig. 6:**
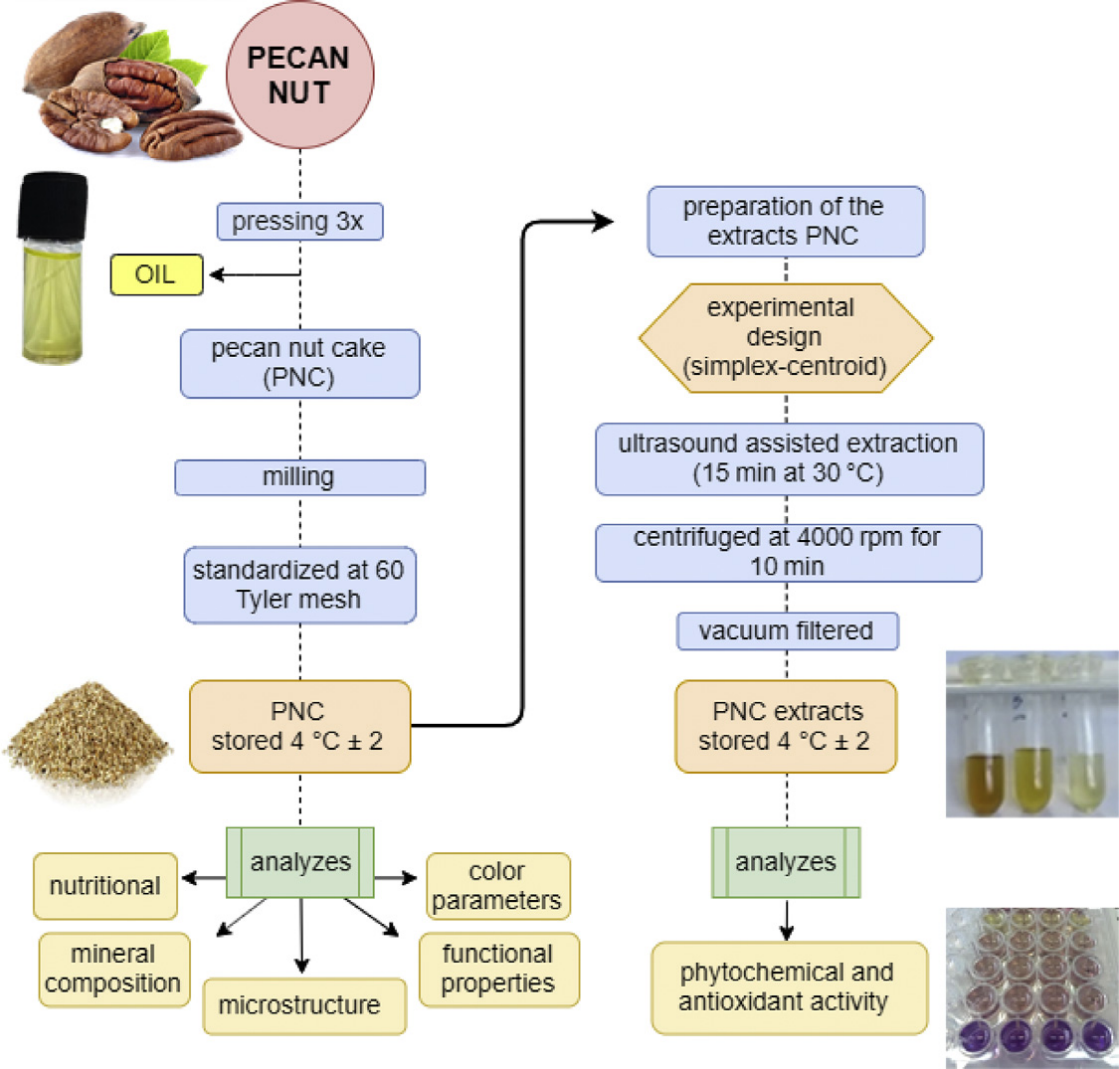
Flowchart of the experimental procedure for obtaining and analyzes for pecan nut cake (PNC) and PNC extracts.

**Fig. 7:**
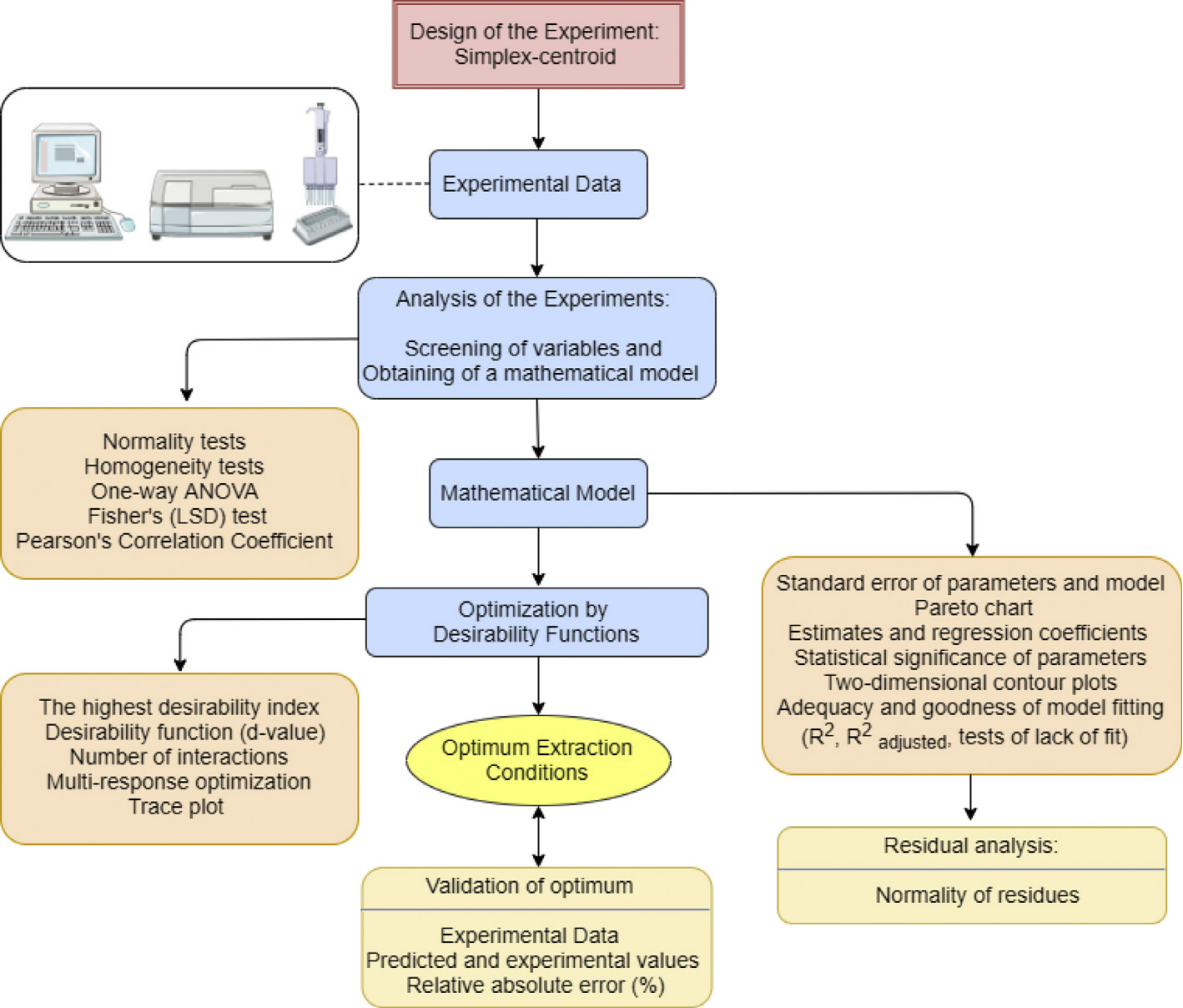
Summary of the statistical procedures used to analyze the dataset.

The manuscript is organized as follows: the data presented in Subsection 1.1*, Screening of variables and obtaining of a mathematical model* (Table 1, Table 2; Fig. 1, Fig. 2, Fig. 3) describe the data on estimates and regression coefficients (Raw data, unadjusted), plots of correlation, Pareto charts, and normality of residuals. In Subsection 1.2, *Optimization by desirability function* (Fig. 4, Fig. 5, and Table 3), we presented data on the multi-response optimization of the mixture of solvents, trace graphs, in addition to the predicted and experimental values for the optimized data. Subsection 2.1, *Preparation of the sample* describes the steps for evaluating the nutritional, mineral, microstructural, and functional properties. Fig. 6 presents a detailed flowchart with the steps of the experimental approach performed. In Subsection 2.2, *Mineral determination parameters* (Table 4), the data on the analytical and instrumental parameters for the analysis of minerals are presented. Subsection 2.3, *Statistical design of the extraction process*, presents the parametric statistical techniques related to the mathematical modeling of processes using experimental design followed by multiple regression analysis, the so-called response surface methodology (RSM). The underlying requirements for assessing the fit, quality and predictability of the generated models are also presented. Fig. 7 describes how the statistical procedure can be used as a tool for analyzing and optimizing the ultrasound-assisted extraction process of the phytochemical and antioxidant content of PNC using a simplex centroid mixture design.

**Table 1:** Raw data (unadjusted), estimates coefficients for modeling the effects of different solvents on the extraction of pecan nut cake.

Parameters	Coefficient estimates	Standard error	t-value	p-value	Confidence	
					−95%	+95%
Total phenolic compounds (TPC) (mg GAE 100 g^−1^)						
(A)Ethanol	1447.098	264.514	5.471	0.001	821.622	2072.574
(B)Water	470.083	187.420	2.508	0.041	26.905	913.262
(C)Acetic Acid	119.121	264.511	0.450	0.666	−506.349	744.591
AB	3238.899	1239.946	2.612	0.035	306.893	6170.905
AC	50.761	1062.360	0.048	0.963	−2461.320	2562.843
BC	8475.452	993.458	8.531	0.000	6126.297	10824.607
ABC	12721.600	6620.827	1.921	0.096	−2934.169	28377.369
AB(A-B)	5736.398	3752.320	1.529	0.170	−3136.428	14609.223
AC(A-C)	−5231.782	3406.511	−1.536	0.168	−13286.900	2823.335
R^2^	0.950					
Adjusted R^2^	0.893					
Condensed tannins (CT) (mg CE 100 g^−1^)						
(A)Ethanol	899.269	91.595	9.818	0.000	682.680	1115.858
(B)Water	567.562	64.900	8.745	0.000	414.099	721.025
(C)Acetic Acid	824.919	91.595	9.006	0.000	608.332	1041.505
AB	2289.640	429.366	5.333	0.001	1274.350	3304.930
AC	−1108.194	367.872	−3.012	0.020	−1978.073	−238.314
BC	60.644	344.013	0.176	0.865	−752.817	874.105
ABC	−15553.60	2292.649	−6.784	0.000	−20974.858	−10132.353
AB(A-B)	5075.161	1299.347	3.906	0.006	2002.695	8147.628
AC(A-C)	2420.822	1179.601	2.052	0.079	−368.490	5210.134
R^2^	0.962					
Adjusted R^2^	0.917					
Reducing potential of the hydrophilic compounds (RPHC) (mg GAE 100 g^−1^)						
(A)Ethanol	642.740	89.180	7.207	0.000	431.861	853.618
(B)Water	192.160	63.188	3.041	0.019	42.743	341.577
(C)Acetic Acid	142.316	89.180	1.596	0.155	−68.561	353.192
AB	2261.903	418.046	5.411	0.001	1273.382	3250.424
AC	1038.074	358.173	2.898	0.023	191.130	1885.018
BC	5469.678	334.943	16.330	0.000	4677.665	6261.692
ABC	8887.079	2232.201	3.981	0.005	3608.764	14165.395
AB(A-B)	4890.098	1265.088	3.865	0.006	1898.639	7881.556
AC(A-C)	−4497.505	1148.499	−3.916	0.006	−7213.274	−1781.736
R^2^	0.987					
Adjusted R^2^	0.972					
2,2-diphenyl-1-picrylhydrazyl (DPPH) (% scavenging activity)						
(A)Ethanol	62.520	1.960	31.901	0.000	57.886	67.154
(B)Water	35.489	1.389	25.557	0.000	32.206	38.773
(C)Acetic Acid	11.783	1.960	6.012	0.001	7.149	16.417
AB	100.466	9.187	10.936	0.000	78.743	122.190
AC	23.026	7.871	2.925	0.022	4.414	41.639
BC	172.663	7.361	23.458	0.000	155.258	190.068
ABC	−28.336	49.054	−0.578	0.582	−144.332	87.659
AB(A-B)	−11.945	27.801	−0.430	0.680	−77.684	53.795
AC(A-C)	−245.523	25.239	−9.728	0.000	−305.204	−185.842
R^2^	0.994					
Adjusted R^2^	0.986					
Total reducing capacity (TRC) (mg QE 100 g^−1^)						
(A)Ethanol	1578.948	304.157	5.191	0.001	859.731	2298.165
(B)Water	89.201	215.509	0.414	0.691	−420.398	598.800
(C)Acetic Acid	389.974	304.154	1.282	0.241	−329.236	1109.184
AB	4268.541	1425.779	2.994	0.020	897.110	7639.972
AC	−1082.168	1221.578	−0.886	0.405	−3970.740	1806.404
BC	3342.757	1142.350	2.926	0.022	641.530	6043.984
ABC	−2751.594	7613.103	−0.361	0.728	−20753.722	15250.533
AB(A-B)	2102.784	4314.687	0.487	0.641	−8099.828	12305.397
AC(A-C)	−8673.913	3917.051	−2.214	0.062	−17936.266	588.439
R^2^	0.854					
Adjusted R^2^	0.688					

* GAE: Gallic acid equivalent; CE: Catechin equivalent; QE: Quercetin equivalent.

**Table 2:** Raw data (unadjusted), regression coefficients for modeling the effects of different solvents on the extraction of pecan nut cake.

Parameters	Regression coefficient	Standard error	t-value	p-value	Confidence	
					−95%	+95%
Total phenolic compounds (TPC) (mg GAE 100 g^−1^)						
(A)Ethanol	57.884	10.581	5.471	0.001	32.865	82.903
(B)Water	18.803	7.497	2.508	0.041	1.076	36.530
(C)Acetic Acid	4.765	10.580	0.450	0.666	−20.254	29.784
AB	5.182	1.984	2.612	0.035	0.491	9.873
AC	0.081	1.700	0.048	0.963	−3.938	4.101
BC	13.561	1.590	8.531	0.000	9.802	17.319
ABC	0.814	0.424	1.921	0.096	−0.188	1.816
AB(A-B)	0.367	0.240	1.529	0.170	−0.201	0.935
AC(A-C)	−0.335	0.218	−1.536	0.168	−0.850	0.181
R^2^	0.950					
Adjusted R^2^	0.893					
Condensed tannins (CT) (mg CE 100 g^−1^)						
(A)Ethanol	35.971	3.664	9.818	0.000	27.307	44.634
(B)Water	22.702	2.596	8.745	0.000	16.564	28.841
(C)Acetic Acid	32.997	3.664	9.006	0.000	24.333	41.660
AB	3.663	0.687	5.333	0.001	2.039	5.288
AC	−1.773	0.589	−3.012	0.020	−3.165	−0.381
BC	0.097	0.550	0.176	0.865	−1.205	1.399
ABC	−0.995	0.147	−6.784	0.000	−1.342	−0.648
AB(A-B)	0.325	0.083	3.906	0.006	0.128	0.521
AC(A-C)	0.155	0.075	2.052	0.079	−0.024	0.333
R^2^	0.962					
Adjusted R^2^	0.917					
Reducing potential of the hydrophilic compounds (RPHC) (mg GAE 100 g^−1^)						
(A)Ethanol	25.710	3.567	7.207	0.000	17.274	34.145
(B)Water	7.686	2.528	3.041	0.019	1.710	13.663
(C)Acetic Acid	5.693	3.567	1.596	0.155	−2.742	14.128
AB	3.619	0.669	5.411	0.001	2.037	5.201
AC	1.661	0.573	2.898	0.023	0.306	3.016
BC	8.751	0.536	16.330	0.000	7.484	10.019
ABC	0.569	0.143	3.981	0.005	0.231	0.907
AB(A-B)	0.313	0.081	3.865	0.006	0.122	0.504
AC(A-C)	−0.288	0.074	−3.916	0.006	−0.462	−0.114
R^2^	0.987					
Adjusted R^2^	0.972					
2,2-diphenyl-1-picrylhydrazyl (DPPH) (% scavenging activity)						
(A)Ethanol	2.501	0.078	31.901	0.000	2.315	2.686
(B)Water	1.420	0.056	25.557	0.000	1.288	1.551
(C)Acetic Acid	0.471	0.078	6.012	0.001	0.286	0.657
AB	0.161	0.015	10.936	0.000	0.126	0.196
AC	0.037	0.013	2.925	0.022	0.007	0.067
BC	0.276	0.012	23.458	0.000	0.248	0.304
ABC	−0.002	0.003	−0.578	0.582	−0.009	0.006
AB(A-B)	−0.001	0.002	−0.430	0.680	−0.005	0.003
AC(A-C)	−0.016	0.002	−9.728	0.000	−0.020	−0.012
R^2^	0.994					
Adjusted R^2^	0.986					
Total reducing capacity (TRC) (mg QE 100 g^−1^)						
(A)Ethanol	63.158	12.166	5.191	0.001	34.389	91.927
(B)Water	3.568	8.620	0.414	0.691	−16.816	23.952
(C)Acetic Acid	15.599	12.166	1.282	0.241	−13.169	44.367
AB	6.830	2.281	2.994	0.020	1.435	12.224
AC	−1.731	1.955	−0.886	0.405	−6.353	2.890
BC	5.348	1.828	2.926	0.022	1.026	9.670
ABC	−0.176	0.487	−0.361	0.728	−1.328	0.976
AB(A-B)	0.135	0.276	0.487	0.641	−0.518	0.788
AC(A-C)	−0.555	0.251	−2.214	0.062	−1.148	0.038
R^2^	0.854					
Adjusted R^2^	0.687					

* GAE: Gallic acid equivalent; CE: Catechin equivalent; QE: Quercetin equivalent.

**Table 3:** Experimental and predicted values for the optimized of the PNC.

Response variable	Experimental values					Predicted mean value	−95% Prediction	+95% Prediction
	R1	R2	R3	Mean ± SD	Relative absolute error (%)^a^			
TPC (mg GAE 100 g^−1^)	1947.82	1908.41	1906.51	1920.91 ± 23.3	0.01	1921.02	1194.96	2647.08
CT (mg CE 100 g^−1^)	1180.23	1209.18	1196.15	1195.19 ± 14.5	20.79	1443.69	1142.12	1745.26
RPHC (mg GAE 100 g^−1^)	1227.73	1194.17	1180.91	1200.94 ± 24.13	8.62	1304.42	1016.42	1592.42
DPPH (% of inhibition)	69.12	69.47	70.14	69.58 ± 0.52	2.62	71.41	65.08	77.73
TRC (mg QE 100 g^−1^)	1734.01	1707.25	1660.45	1700.57 ± 37.23	10.77	1883.75	901.49	2866.01

R1, R2, and R3 = replicate; SD = standard deviation.

a Relative absolute error was calculated as (%) = [(experimental mean value − predicted mean value)/predicted mean value × 100].

**Table 4:** Analytical and instrumental parameters for the analysis of mineral by flame atomic absorption spectrometry (F-AAS) and Flame Atomic Emission Spectrometry (F-AES).

Determination	Method	Sample weight (g)	Angular coefficient	Linear coefficient	Wavelength (λ) (nm)	Analytical range
Calcium	F-AAS	5.0 ± 0.1	0.06486	0.00438	422.67	1.00–5.00 (mg kg^−1^)
Copper	F-AAS	5.0 ± 0.1	0.15272	0.000204	324.80	0.25–1.60 (mg kg^−1^)
Cobalt	F-AAS	5.0 ± 0.1	0.07525	−0.00046	240.73	0.02–0.60 (mg kg^−1^)
Zinc	F-AAS	5.0 ± 0.1	0.52012	0.02558	213.86	0.10–1.50 (mg kg^−1^)
Magnesium	F-AAS	5.0 ± 0.1	1.04741	−0.01038	285.21	0.10–0.30 (mg kg^−1^)
Manganese	F-AAS	5.0 ± 0.1	0.20836	0.0007	279.50	0.05–0.75 (mg kg^−1^)
Iron	F-AAS	5.0 ± 0.1	0.09185	0.00317	248.30	0.50–3.00 (mg kg^−1^)
Sodium	F-AES	3.0 ± 0.1	1.0557	0.2286	589.00	1.00–10.00 (mg L^−1^)
Potassium	F-AES	3.0 ± 0.1	1.0257	0.3619	710.00	1.00–10.00 (mg L^−1^)

### 1.1 Screening of variables and obtaining of a mathematical model

The data regarding the content of phytochemicals and antioxidant activity obtained in the ultrasound-assisted extraction using the mixtures of ethanol, acetic acid, and water used for calculating the following statistics were reported in our previous work [1]. The data used to propose mathematical equations that explained the effects of each type of solvents on the phytochemicals and antioxidant activity of extracts are reported in Table 1, Table 2, and Fig. 1, Fig. 2, Fig. 3.

### 1.2 Optimization by desirability function

The data regarding the multiple linear regression based on the RSM was used to propose the simultaneous optimization that explained the effects of each type of solvents on the phytochemicals and antioxidant activity of extracts (Fig. 4, Fig. 5). The data used for calculating for the best option to obtain a mixture with maximized antioxidant capacity are reported in Table 3.

## 2 Experimental design, materials, and methods

### 2.1 Preparation of the sample

The pecan nut sample was processed as reported by Maciel et al. [1]. After the oil removal, the obtained cake was evaluated for its nutritional and mineral compositions, microstructure, and functional properties. Then, the sample was extracted with the aid of an ultrasound system to obtain antioxidant-rich extracts according to an experimental design [1]. Finally, these extracts were evaluated for determining the total phenolic compounds, condensed tannins, and antioxidant activity. Fig. 6 shows a schematic diagram of the analyses performed for obtaining the pecan nut cake (PNC) and its corresponding extracts.

### 2.2 Parameters for minerals determination

A total of 9 elements were evaluated (calcium, magnesium, sodium, and potassium) and 5 trace elements (zinc, manganese, copper, iron, cobalt). The analytical and instrumental parameters are specified in Table 4.

### 2.3 Statistical design of the extraction process

All the analyses were conducted in triplicate, and the data expressed as original replicates or the mean ± standard deviation. To screen the variables and obtain a mathematical model, we firstly evaluated the significant statistical differences using one-way ANOVA, followed by a Fisher LSD test (p ≤ 0.05) for parametric and homoscedastic data. The normality of the data was checked by the Shapiro-Wilk test, while the Brown-Forsythe test was used for homoscedasticity. Linear correlation analysis was performed to verify the degree of association between responses and regression analysis. Linear correlations were calculated and expressed by Pearson's correlation coefficient (r), where *p* values below 5% were considered significant. Correlation strengths were evaluated according to the following criteria: perfect (r = 1.0), strong (r < 1.0 and ≥0.8), moderate (r < 0.80 and ≥0.50), weak (r < 0.50 and ≥0.10) and very weak (r < 0.10) [2].

The RSM was applied to estimate the effects of different solvents on the content of phytochemicals and the antioxidant activity of PNC. RSM was also applied for modeling the regression coefficients as a function of the variables (types of solvents). The analysis of variance of the models was calculated, and the effects and regression coefficients of the linear, quadratic, and cubic terms were determined. Non-significant regression coefficients (p ≥ 0.05) were discarded, and data were reevaluated to obtain the final model for each parameter. The statistical quality of the proposed models was evaluated by the percentage of variability explained by the coefficient of determination (R^2^), the adjusted coefficient of determination (R^2^_adj_), and the significance of the model (p ≤ 0.05). The P_lack of fit_ value was used to verify the adequacy of the model, where models with P_lack of fit_ > 0.05 indicate that it can adequately adjust to the experimental data. In addition, a confidence interval of ±95% was also measured for each effect. Regression coefficients were then used to generate Pareto charts and two-dimensional contour plots for each response. The residuals plots were examined for all response variables, for obvious patterns (predicted vs. experimental data) and formally tested for normality using the Kolmogorov-Smirnov test [3].

After modeling the responses, the optimization by desirability function steps was performed. The maximization of TPC, CT, and antioxidant activity of the PNC was predicted by the desirability function and *d*-value, which is a measure of how much the proposed formulation conforms to the main goal of the optimization obtained. A total of 80 interactions were run to provide the best condition for optimization. Then, the trace plot and multi-response optimization graph were generated for each response variable. Finally, the experimental validation of the values obtained with the optimization against the predicted values was performed. The Statistica software v. 10.0 (StatSoft Inc., USA), Microsoft Office Excel® v. 2016 (Microsoft Inc., USA), Action v.2.9 (Statcamp, Brazil) were used for data processing. A summary of all the steps that were used to apply the statistical tools is presented in Fig. 7.
